# Public opinion about the health care system in Armenia: findings from a cross-sectional telephone survey

**DOI:** 10.1186/s12913-020-05863-6

**Published:** 2020-11-03

**Authors:** Tsovinar Harutyunyan, Varduhi Hayrumyan

**Affiliations:** 1 Tsovinar Harutyunyan, Turpanjian School of Public Health, American University of Armenia, 40 Marshal Baghramyan Ave., 0019 Yerevan, Armenia; 2 Varduhi Hayrumyan, Turpanjian School of Public Health, American University of Armenia, 40 Marshal Baghramyan Ave., 0019 Yerevan, Armenia

## Abstract

**Background:**

Few studies have examined public opinion about the health care system in the former Soviet region. The objective of our study was to evaluate the population’s satisfaction with the health care system and identify factors associated with it in Armenia.

**Methods:**

We conducted a cross-sectional telephone survey among 576 adult residents of the capital Yerevan using Random Digit Dialing technique. Simple and multivariate logistic regression explored associations between potential determinants and satisfaction.

**Results:**

A substantial proportion of respondents (45.5%) were dissatisfied or very dissatisfied with the health system. About 49% of respondents negatively evaluated the ability of the system to provide equal access to care. About 69% of respondents thought that the responsibility for an individual’s health should be equally shared between the individual and the government or that the government’s share should be larger. The adjusted odds of satisfaction were higher among individuals with better health status, those who positively rated equal access and respect to patients in the system, those thinking that the responsibility for health should be equally shared between the individual and the government, and those who tended to trust the government.

**Conclusions:**

This study enriched our understanding of factors that shape the population’s satisfaction with the health care system in different cultural and political environments. We recommend further exploration of public opinion about those system attributes that are not directly linked to patient experiences with care, but might be equally important for explaining the phenomenon of satisfaction.

**Supplementary Information:**

**Supplementary information** accompanies this paper at 10.1186/s12913-020-05863-6.

## Background

The importance of assessing patient satisfaction as an essential component of health care outcomes has been long recognized [1]. While most of the studies in this field focus exclusively on patients’ experiences with health care services, the research that explores population’s satisfaction with health care system, in general, is relatively limited [2, 3]. The literature addressing both patient and general population satisfaction in the former Soviet Union countries is particularly scarce [3, 4].

In Armenia, as well as in many other former Soviet countries, the health care system destabilized following independence in 1991, leading to a substantial decline in access to and quality of health care [4–6]. The governments in the post-Soviet region faced an increasing gap between their commitments to free health care and fiscal constraints [3]. The countries have substantially varied in terms of trajectories of health system change, specific reform instruments, and available resources [7]. In Armenia, the period of destabilization has been followed by substantive reforms, including decentralizing service provision to regional governments, separation of purchasing and provider functions, and privatization of services [8, 9]. The current health system includes independent, self- or mixed-financed health services that provide state and private services [9]. According to the recent estimates, total health expenditure as a percent of Gross Domestic Product (GDP) in Armenia was 10.36% in 2017 [10], while public expenditure on health care remained below 2% of GDP in the after-independence period [9].

The government has attempted to improve access to care through several programs. The Basic Benefit Package (BBP), which was introduced in 1996, specifies services that are either fully or partially funded for socially vulnerable groups and the entire population [8, 9]. As of 2016, the package included free primary care, maternity services, sanitary-epidemiological services, and treatment for around 200 socially significant diseases for the entire population, as well as hospital care for children under the age of 7, and mandatory basic health insurance coverage for public sector employees [9].

In 2006, to ensure access to free and quality maternity services for all women, the Government of Armenia introduced the Obstetric Care State Certificate. The reform substantially reduced out-of-pocket payments for deliveries among the Armenian women [9]. Another reform introduced in 2011 was the Child Health State Certificate which was also successful in terms of reducing informal payments, improving access to and affordability of pediatric health care services eventually leading to greater patient satisfaction [9]. In 2012, the Armenian Government introduced the Social Package for government and public sector employees who have been given an opportunity to purchase private health insurance to cover some health care needs [9].

The periodical review of BBP with the expansion or reduction of services and/or population groups covered led to certain a level of confusion among service users and health care providers [9]. The limited scope of BBP did not allow solving the problem of limited access to services. The proportion of out-of-pocket payments in total health expenditure was 84.35% in 2017 [11], suggesting enduring problems in health system financing in Armenia [9].

Despite continuous gaps in the system, the study conducted in nine former Soviet countries has shown a high level of satisfaction with the system in Armenia (53.8%) in 2010 [3]. A 24.3% increase in overall satisfaction levels from 2001 has also been documented [3]. It was assumed that this change (similar to changes in many other post-Soviet countries) might reflect general improvements in living conditions, a better understanding of how the reorganized health systems work, as well as adjusted expectations from the Soviet era and decreasing proportions of respondents who have experienced the Soviet care [3].

The above-mentioned study has not provided a detailed analysis of factors influencing satisfaction with health care in Armenia. The direct exploration of the Armenian population’s expectations, beliefs and values that might shape the satisfaction levels could therefore be a valuable contribution to the limited existing evidence in the field.

The investigation of public opinion can foster a clearer communication of what citizens expect from the system and what can be reasonably provided, and serve as a groundwork for public inclusion in decision-making processes [3, 12]. While studies focusing specifically on patient satisfaction with received services explore direct experiences of the system users, public satisfaction research includes non-users as well as users of health care, reflects assessments from a more general and more societal point of view, and is shaped by wider factors including reports of other users’ experiences and media influences [13].

The primary aim of this study was to assess the Armenian population’s satisfaction level with the health care system and explore associated factors.

## Methods

We conducted a cross-sectional telephone survey among the adult population of capital Yerevan, where roughly 37% of Armenia’s population are located [14]. To be included in the survey the respondents had to be permanent residents of Armenia in the last 5 years and able to speak Armenian.

The survey respondents were sampled using Stratified Random Sampling proportionate to size of the population living in each district of Yerevan. Random Digit Dialing (RDD) of landline phone numbers was used to enroll the participants [15]. Repeated dialing continued until reaching the intended sample size of 576 complete interviews. Prior to fielding, the researchers conducted an interviewer training. The data collection was conducted by three interviewers during July and August 2016. The interviewers conducted calls during different times of the day to ensure the representativeness of the sample. All respondents were explained the purpose of the survey and provided oral informed consent prior to the interview start. In case of refusal, the interviewers attempted to understand the reasons for refusal and recorded them in the survey journal form. If a lack of time was mentioned as a reason, the interviewers attempted to arrange the interview at a more suitable time.

We used a structured questionnaire developed based on the tools from similar studies conducted internationally and in Armenia, located through extensive literature search [3, 4, 16–19].

The questionnaire was pre-tested prior to data collection and contained domains on satisfaction with health care system, health status and utilization of health care services, awareness about recent health care reforms, the relative importance of health system characteristics, government’s responsibility for the health of individuals and socio-demographic characteristics of participants. In total, the questionnaire included 27 survey questions.

The dependent variable was the respondents’ satisfaction with the health care system in Armenia. Satisfaction was measured by a Likert scale type question with balanced response options of “very satisfied”, “satisfied”, “neither satisfied nor dissatisfied”, “dissatisfied”, and “very dissatisfied”. The independent variables included socio-demographic characteristics, self-rated health status, use of health care services in the last 12 months, awareness about governmental reforms to improve access to care, evaluation of six system attributes (equal access to care, professional qualifications of providers, quality of basic amenities, respect to patients, and ability to choose a provider) in the current system, opinion about the role of the government in preserving individual’s health, and trust in the government.

The data was entered, cleaned and analyzed using SPSS 22 and STATA 13 software (SPSS Inc., Chicago, IL, USA, and Stata Corporation, College Station, Texas, USA). Descriptive analysis was followed by bivariate and multivariate logistic regression to reveal crude and independent associations between independent and dependent variables. The responses of those who chose the neutral option of the satisfaction variable were excluded from the binary outcome used in bivariate and multivariate analysis.

The Institutional Review Board of the American University of Armenia approved the study (#AUA-2016-017). Oral informed consent was obtained from all participants.

## Results

Overall, 5851 call attempts were made to reach the study participants, resulting in 2030 contacts. Out of the contacted respondents, 30.8% did not meet the inclusion criteria. About 57% of the remaining respondents refused to participate in the study, with the most commonly cited reason being having no time. Overall, 576 completed interviews were obtained. There were twenty-seven incomplete interviews. Table 1 describes socio-demographic characteristics of the respondents.

**Table 1:** Socio-demographic characteristics of the study participants

Variables	% (n)
Age, years	
Mean	45.22
SD	16.31
Median	43
Range	18–84
Age categories, % (n)	
18–35	34.20 (197)
36–65	52.78 (304)
> 66	13.02 (75)
Gender, % (n)	
Male	16.67 (96)
Female	83.33 (480)
Educational level, % (n)	
School (8 years or less)	1.91 (11)
School (10 years)	13.74 (79)
Professional technical (10–13)	25.04 (144)
Institute/ University	58.09 (334)
Post-graduate	1.22 (7)
Employment status, % (n)	
Employed (including self-employed)	39.58 (228)
Unemployed	41.49 (239)
Retired	15.10 (87)
Student	3.82 (22)
Marital status, % (n)	
Single	17.16 (98)
Married	68.65 (392)
Widowed	10.16 (58)
Divorced/Separated	4.03 (23)
Family monthly spending on average, % (n)	
Less than 50,000 drams	4.20 (18)
From 50,000–100,000 drams	23.78 (102)
From 100,001–200,000 drams	38.69 (166)
From 200,001–300,000 drams	21.68 (93)
Above 300,000 drams	11.66 (50)
Household members	
Mean	3.98
SD	1.82

Approximately 46% of the respondents were either dissatisfied or very dissatisfied with the health care system as opposed to 25.4% who were satisfied or very satisfied (Table 2).

**Table 2:** Satisfaction with healthcare system, health status, use of healthcare services and trust in the government among study participants

Variables	Total %(***n*** = 576)	Male 16.67% (***n*** = 96)	Female 83.33% (***n*** = 480)	***P***-value
In general, would you say you are very satisfied, satisfied, neither satisfied nor dissatisfied, dissatisfied, or very dissatisfied with healthcare in Armenia?				
Very satisfied	1.39 (8)	3.13 (3)	1.04 (5)	0.156
Satisfied	23.96 (138)	27.08 (26)	23.33 (112)	
Neither satisfied nor dissatisfied	29.17 (168)	34.38 (33)	28.13 (135)	
Dissatisfied	31.42 (181)	25.00 (24)	32.71 (157)	
Very dissatisfied	14.06 (81)	10.42 (10)	14.79 (71)	
In general, would you say your health is …				
Excellent	6.78 (39)	15.63 (15)	5.01 (24)	< 0.001
Very good	6.09 (35)	4.17 (4)	6.47 (31)	
Good	28.17 (162)	39.58 (38)	25.89 (124)	
Fair	44.00 (253)	34.38 (33)	45.93 (220)	
Poor	14.96 (86)	6.25 (6)	16.70 (80)	
Have you used any health care services in the last 12 months?				
Yes	66.15 (381)	47.92 (46)	69.79 (335)	< 0.001
No	33.85 (195)	52.08 (50)	30.21 (145)	
What was the reason for not using health care services in the last 12 months?				
There was no need	48.45 (94)	68.00 (34)	41.67 (60)	0.001
Could not afford the services	31.44 (61)	20.00 (10)	35.42 (51)	0.043
Absence of time	8.25 (16)	6.00 (3)	9.03 (13)	0.503
Do not trust doctors	18.56 (36)	22.00 (11)	17.36 (25)	0.467
Fear of diagnosis	1.55 (3)	0.00 (0)	2.08 (3)	0.304
Other	8.25 (16)	4.00 (2)	9.72 (14)	0.205
In general, would you say you are very satisfied, satisfied, neither satisfied nor dissatisfied, dissatisfied, or very dissatisfied with the last healthcare services you received in the last 12 months?				
Very satisfied	15.53 (59)	21.74 (10)	14.67 (49)	0.481
Satisfied	50.79 (193)	52.17 (24)	50.60 (169)	
Neither satisfied nor dissatisfied	14.47 (55)	15.22 (7)	14.37 (48)	
Dissatisfied	12.89 (49)	8.70 (4)	13.47 (45)	
Very dissatisfied	6.32 (24)	2.17 (1)	6.89 (23)	
In general, would you say that you tend to trust or not to trust the Armenian government?				
Tend to trust	20.95 (101)	73.75 (59)	19.90 (80)	0.203
Tend not to trust	79.05 (381)	26.25 (21)	80.10 (322)	

The respondents mostly characterized their health as fair (44%) or good (28.2%). About 66% of the respondents used health care services in the last 12 months. Approximately 16% of them were very satisfied and 50.8% were satisfied with the services received (Table 2). The major reasons for not using health services included having no need (48.5%) and an inability to pay for the services (31.4%).

The overwhelming majority of the respondents knew about major reforms carried out by the government to improve access to care (78 to 87% awareness about each program). However, most of them (53.4%) thought that the listed reforms did not help to improve access to care for Armenian citizens. When asked to explain why the reforms were not helpful, most of them said that the improvements did not work as expected (60.7%). Equal proportions thought that the improvements apply to narrow population groups (34.7%) or to limited services (34.7%).

The respondents were asked to rate the state of several health system attributes in the Armenian system (Table 3). The ability to choose a provider was the characteristic most positively evaluated by the respondents with 71.8% rating it as good or very good. The characteristic that was negatively evaluated by the highest proportion of respondents was equal access to care (49.1% rating it as bad or very bad) (Table 3).

**Table 3:** Participants’ rating of selected attributes of the health care system in Armenia

Attribute	Total %(***n*** = 576)	Male 16.67% (***n*** = 96)	Female 83.33% (***n*** = 480)	***P***-value
a. Equal access to care for all citizens				
Very bad	20.70 (119)	12.50 (12)	22.34 (107)	0.045
Bad	28.35 (163)	39.58 (38)	26.10 (125)	
Neither bad nor good	40.00 (230)	35.42 (34)	40.92 (196)	
Good	10.09 (58)	11.46 (11)	9.81 (47)	
Very good	0.87 (5)	1.04 (1)	0.84 (4)	
b. Modern medical equipment				
Very bad	1.22 (7)	2.08 (2)	1.04 (5)	0.308
Bad	6.09 (35)	7.29 (7)	5.85 (28)	
Neither bad nor good	39.13 (225)	45.83 (44)	37.79 (181)	
Good	41.74 (240)	37.50 (36)	42.59 (204)	
Very good	11.83 (68)	7.29 (7)	12.73 (61)	
c. Professional qualifications of providers				
Very bad	3.65 (21)	3.13 (3)	3.76 (18)	0.588
Bad	9.91 (57)	10.42 (10)	9.81 (47)	
Neither bad nor good	40.70 (234)	46.88 (45)	39.46 (189)	
Good	38.78 (223)	35.42 (34)	39.46 (189)	
Very good	6.96 (40)	4.17 (4)	7.52 (36)	
d. Respect to patients				
Very bad	8.89 (51)	7.29 (7)	9.21 (44)	0.386
Bad	15.68 (90)	10.42 (10)	16.74 (80)	
Neither bad nor good	41.11 (236)	48.96 (47)	39.54 (189)	
Good	27.18 (156)	26.04 (25)	27.41 (131)	
Very good	7.14 (41)	7.29 (7)	7.11 (34)	
e. Ability to choose a provider				
Very bad	1.04 (6)	1.04 (1)	1.04 (5)	0.018
Bad	2.26 (13)	1.04 (1)	2.51 (12)	
Neither bad nor good	24.87 (143)	38.54 (37)	22.13 (106)	
Good	48.35 (278)	40.63 (39)	49.90 (239)	
Very good	23.48 (135)	18.75 (18)	24.43 (117)	
f. Quality of basic amenities (cleanliness, ventilation, quality of food, number of patients in the room, etc.)				
Very bad	1.91 (11)	0.00 (0)	2.30 (11)	0.365
Bad	8.00 (46)	6.25 (6)	8.35 (40)	
Neither bad nor good	54.09 (311)	61.46 (59)	52.61 (252)	
Good	29.57 (170)	27.08 (26)	30.06 (144)	
Very good	6.43 (37)	5.21 (5)	6.68 (32)	

The largest proportion of the respondents thought that the responsibility for health should be shared between the individual and the government, but the government’s responsibility is larger (34.7%). A similar proportion (34.2%) thought that there should be an equal share of responsibility. Approximately 17% thought that the individual’s responsibility should be larger, 10% thought that it is solely the government’s responsibility, and 4% thought that it is solely the individual’s responsibility.

Participants were asked about whether they tend to trust or not to trust the government. About 66% said that they tend not to trust the government, 17.5% said that they tend to trust it, and 16.3% refused to answer.

### Bivariate and multivariate analysis

All variables which were associated with satisfaction at *p* < 0.1 level in bivariate analysis were entered into the adjusted logistic regression model (Table 4). The variables included participants’ age, gender, employment status, monthly family spending, health status, ratings of equal access to care, professional qualifications of providers, respect to patients, and quality of basic amenities in the current system, opinion on whether the responsibility for health lies with the government or the individual, and trust in government.

**Table 4:** Adjusted associations between satisfaction with Armenian healthcare system and independent variables

Variable	OR (95% CI)	***p***-value
**Age** (years)	0.99 (0.96–1.01)	0.240
**Gender**		
Male	1.00	
Female	0.62 (0.26–1.46)	0.275
**Family monthly spending on average** (AMD)		
Less than 100,000	1.00	
From 100,001 - 200,000	1.22 (0.57–2.59)	0.606
Above 200,001	1.40 (0.62–3.19)	0.421
**Number of household members**	1.05 (0.88–1.26)	0.564
**Health status**		
Excellent/Very good	1.00	
Good	0.43 (0.15–1.26)	0.125
Fair/Poor	0.25 (0.09–0.74)	0.012
**Rating of system attributes in Armenian health care system**		
a. Equal access to care for all citizens		
Very bad/Bad	1.00	
Neither bad nor good	1.62 (0.85–3.10)	0.144
Good/Very good	5.69 (1.91–16.88)	0.002
c. Professional qualifications of providers		
Very bad/Bad	1.00	
Neither bad nor good	2.08 (0.73–5.90)	0.171
Good/Very good	1.97 (0.69–5.63)	0.204
d. Respect for patients		
Very bad/Bad	1.00	
Neither bad nor good	1.42 (0.64–3.15)	0.388
Good/Very good	2.69 (1.10–6.57)	0.030
d. Quality of basic amenities		
Very bad/Bad	1.00	
Neither bad nor good	1.25 (0.42–3.75)	0.691
Good/Very good	1.40 (0.43–4.53)	0.571
**Do you think it is the individual’s responsibility to preserve his/her own health or it is the responsibility of the government?**		
Government’s responsibility/ Shared, but the government’s responsibility is lager	1.00	
Individual responsibility/Shared, but the individual’s responsibility is larger	1.39 (0.61–3.20)	0.436
Equal share of responsibility	1.93 (0.97–3.83)	0.062
**In general, would you say that you tend to trust or not to trust the Armenian government?**		
Tend to trust	1.00	
Tend not to trust	0.47 (0.23–0.96)	0.038

Number of observations = 269

In the adjusted analysis, those who reported fair/poor health status were less likely to be satisfied with the health care system as compared to those who had excellent or very good status (OR = 0.25, 95% CI: 0.09–0.74, *p* = 0.012). Positive rating of equal access to care in the Armenian health care system was associated with substantially higher odds of satisfaction (OR = 5.69, 95% CI: 1.91–16.88, *p* = 0.002) as compared to negative assessments. Similarly, those who positively evaluated respect to patients in the system were 2.69 times more likely to be satisfied with it (95% CI: 1.10–6.57, *p* = 0.030) than those who had not.

Those who thought that the responsibility for health of the individual is equally shared between the individual and the government were more likely to be satisfied with the health care system than those who thought that it is solely the government’s responsibility or that the responsibility is shared but the government’s share is larger at the marginal level of significance with the OR of 1.93 (95% CI: 0.97–3.83, *p* = 0.062).

Not trusting the government was associated with substantially lower odds of satisfaction (OR = 0.47, 95% CI: 0.23–0.96, *p* = 0.038).

## Discussion

The purpose of this study was to assess the level of satisfaction with the health care system and explore factors associated with it in Armenia. A substantial proportion of respondents in our sample (45.5%) reported being either dissatisfied or very dissatisfied with the system. Only 25.4% were satisfied or very satisfied, while the remaining proportion was neutral. Participants’ health status, ratings of equal access to care and respect to patients in the system, opinion on whether the responsibility for health lies with the government or the individual, and trust in government were found to be associated with satisfaction in the adjusted analysis.

The level of dissatisfaction found in this study closely resembles the one reported in the study which explored patient satisfaction in nine former Soviet countries, where 46.2% of Armenian respondents were either quite dissatisfied or definitely dissatisfied with health care [3]. However, that study reported a substantially higher proportion of those who were satisfied (53.8%) which is most likely due to the different approach to the operationalization of the satisfaction variable. In our study a Likert scale type question with 5 response options including a neutral option was used, while in the mentioned study the respondents were choosing between “definitely dissatisfied”, “quite dissatisfied”, “quite satisfied”, and “definitely satisfied” categories. It is possible therefore that a substantial proportion of those who would otherwise choose the neutral option fell in the “quite satisfied” category in their study. We think that our measurement is more precise in discerning different levels of satisfaction and therefore it might be more accurate in the estimation of the true satisfaction level with the system and more in accordance with the actual system performance [3, 8]. Also, since the responses to the question of satisfaction might be conditioned by culturally specific approaches to processing/expressing dissatisfaction [20], including the neutral category in the assessments of satisfaction might be justified. For example, a study conducted in Japan and the US suggested that the neutral responses in the survey of satisfaction with health care services among Kioto respondents might have been a reflection of the typical Japanese saying “Shikata ga nai” (“it can’t be helped”), signifying the attitude of resignation which one feels when he/she is unable to change anything in a particular situation [20]. Similarly, a common Armenian expression “eli lava” (“it could be worse”) is often used when someone is unhappy with the situation but believes he/she should be satisfied with it because it could be worse.

Interestingly, the rate of satisfaction with the services received in the last 12 months was substantially higher than the overall satisfaction rate with the system recorded in this study (66.3% versus 25.4%, respectively), which is in agreement with the studies conducted in other regions of the world [21]. This discordance is explained by the conceptual difference between the two measures. While the question about satisfaction with the overall system might reflect citizens’ opinions about the organization and financing of the system as a whole, the question about satisfaction with care received in the last 12 months assesses the quality of specific services received [21].

A solid proportion of respondents who have not used health care services in the last 12 months (31.4%) mentioned the inability to pay for health services as the reason for not using them, which is quite close to the findings of previous studies conducted in 2010 and 2016 [19, 22] and signifies a substantial unmet need for health care in the Armenian population.

Although the awareness about major health sector reforms was pretty high, from the perspective of most of the surveyed respondents the reforms did not help to meet their needs. This wide disapproval of the reforms combined with high awareness about them might reflect the actual poor implementation of the reforms in Armenia rather than inadequate communication about them to the public, which has also been alleged by some authors [3, 9].

The highest proportions of respondents in our study thought that responsibility for health should be either equally shared between the individual and the government or that the government’s share should be larger. Our data resemble the findings of the Global Trends 2014 survey in Russia where the majority of the respondents (52%) thought that it is the government’s responsibility to support the healthy lifestyle of the population as opposed to individual responsibility – the highest proportion across 20 surveyed countries [23]. On the contrary, only 12% of the respondents from the US thought that it is the responsibility of the government [23]. The researchers explained these differences in attitude partly by the history of local health care systems. According to other authors, people’s attitudes toward their own health can be shaped by the ideology which encourages freedom and personal responsibility [24]. The high expectations for the governments in the area of health in Armenia and Russia might reflect a shared legacy of the radical approach used in the former Soviet Union where massive state interventions in social and health spheres were typical [24, 25].

Our finding that fair/poor health status is associated with lower satisfaction is in line with the results of studies conducted in the same region and in other countries in Europe [2, 3, 26]. Some authors claim that poorer health might mean higher dependence/more frequent use of health care services and therefore, the assessment of the health care system by those in poor health is very important and potentially different from the assessment of those who have fewer direct contacts with health care [26]. However, in our study, the use of services was shown to be unrelated to satisfaction, which implies that health status exerted its influence outside the context of actual care. Interestingly, there have been reports in the literature that satisfaction with services might be unrelated to the change in health status achieved as a result of care [27]. Instead, how good or bad the patients feel at the moment is more important for their evaluation of care [27]. A study conducted among older patients hypothesized that those with poorer health status become dissatisfied with life in general and convey that stance to other aspects of life, including health care [28]. If that is the case, general dissatisfaction with life could also explain the association between poor health status and dissatisfaction with the overall health care system observed in our study.

Out of six system attributes, only equal access to care for all citizens and respect to patients seemed to influence patients’ satisfaction with the system. Respect for patients is one element of responsiveness, a concept developed by WHO, which refers to how the system performs in non-health aspects [29]. In addition to respect for patients, it also includes dignity, autonomy, prompt attention, quality of basic amenities, access to social support networks, and choice of care providers [29]. While the links between the ratings of most of the responsiveness domains, including respect for patients, and satisfaction have been reported in prior studies [2], to the best of our knowledge no studies have documented the independent association between population’s perception of the system’s ability to ensure equal access to care with the level of satisfaction with the system. We think that this finding necessitates further exploration of those system attributes that are not directly linked to patient experience but might be equally important for explaining the phenomenon of satisfaction across different cultural and socio-economic contexts.

We believe that our finding of higher satisfaction among those respondents who favored equal share of responsibility for individual’s health by the government and the individual as opposed to those who thought it is fully or largely government’s responsibility points out to the importance of studying new ways of measuring patient expectations, embodied with this particular variable in our study. The researchers exploring the concept of satisfaction often use proxy measures for patient expectations such as education, income, age, rurality, and others [2–4]. The need for the development of better measures of patient expectations has been acknowledged previously [2]. In this study, we were able to not only show substantial support in the Armenian public for the government’s involvement in individuals’ health but also to demonstrate how central this expectation is to explaining satisfaction with the health care system. We think that it is important for the governments to have a clear understanding of the current level of expectations that citizens have, even if they are assumed to be shaped by the legacy of the Soviet system which collapsed more than 25 years ago [3] and are mismatched with the existing political agenda and resources.

Trust in government was associated with higher satisfaction in our study. The concept of trust in government includes individuals’ attitudes toward government based on their assessments of how well elected officials and public organizations meet individual expectations [30]. This assessment is influenced by and influences social institutions, economic performance, and political processes. Our finding is in accordance with the results of earlier studies conducted in post-soviet countries, which are thought to have inherited a lack of trust in political systems from Soviet times [3]. These studies explored whether health system satisfaction is associated with trust in political institutions more broadly, including trust in president, government, parliament, courts, army, police and political parties, while our measure included only one general question. It has been claimed that a distrust of government has historically weakened public support for a particular national health plan once it has been proposed and debated in the United States [31]. Our finding further supports the assumption that broader societal factors unrelated to patient experience might play a central role in explaining satisfaction with the health care system [2, 31].

### Limitations

This study has several limitations. First of all, the restriction of the sample to the urban Yerevan population, and having the majority of educated and female respondents in the sample might limit the generalizability of our results. Although the landline phone coverage in Yerevan is substantial [32], the exclusion of mobile phones which are increasingly used by the population in many countries, including Armenia, could have introduced another restriction on the representativeness of the sample [33, 34]. The high non-contact and refusal rates in this study, although comparable to the rates from other telephone and RDD surveys conducted internationally [34–37] and in Armenia [38, 39], limit the representativeness of the sample and further stress the need for future telephone surveys in Armenia to expand the respondent base and modify sampling techniques. Second, given the limitations in the acceptable length of this telephone survey, some of the questions were not explored in depth. For example, trust in the government was explored with one direct question, while asking a series of related questions could have resulted in a more accurate estimate and a lower proportion of those who refused to answer this question. Also, since we used self-reports for the questions about the use of health care services in the last 12 months, a certain level of recall bias might have been present.

Another limitation is the exclusion of responses of those who chose the neutral option of the satisfaction variable from bivariate and multivariate analysis, which decreased the sample size available for the logistic regression analysis.

## Conclusions

This study makes an important contribution to the international literature published on the topic of satisfaction with the health care system, as it helps to improve our knowledge about factors that influence it in different cultural and political environments. In democratic countries public norms and opinions are widely believed to affect policy [17]. However, before any attempts are made to change health policies in Armenia accounting for public preferences, it is necessary to obtain more information about those preferences and the underlying beliefs and values. Our findings provide the policy makers in Armenia and in countries with similar profiles with important insights into values and priorities of the population with regard to health care and stress the importance of broader societal factors unrelated to patient experience in shaping the population’s opinion about the health care system. Given the major transformation of the political landscape and functioning of all social institutions in Armenia in 2018, a follow-up study that would explore corresponding changes in public satisfaction with care is pertinent. Future studies might include qualitative research to validate and explore in-depth the findings of the present study and a nation-wide survey with the inclusion of all provinces of Armenia for better representativeness.

## Supplementary Information

**Additional file 1.** Survey questionnaire.

## Data Availability

The datasets used and analyzed during the current study are available from the corresponding author on reasonable request.

## Abbreviations

BBP: Basic Benefit Package
CI: Confidence Interval
GDP: Gross Domestic Product
OR: Odds Ratio
RDD: Random Digit Dialing
