# Supplementary material for: Public opinion about the health care system in Armenia: findings from a cross-sectional telephone survey
Source: BMC Health Serv Res. 2020 Nov 3;20:1005. doi: 10.1186/s12913-020-05863-6 (PMC7640423; doi:10.1186/s12913-020-05863-6)
Supplement: Supplementary file 1 — Additional file 1. Survey questionnaire. [file 12913_2020_5863_MOESM1_ESM.docx]

**Survey questionnaire (English version)**

**Public opinion about healthcare in Armenia**

**ID___ ___ ___ ___ ___ Start Time:____/____**

**Date (DD/MM/YY)_____/____/______ End Time: ____/____**

***Screening questions:***

| 01. | Your age at your last birthday? | ____years |
| --- | --- | --- |
| 02. | Have you permanently resided in Armenia in the last 5 years (leaving the country only periodically and for short periods)? | □ 1) Yes ***→go to the next question***  □ 0) No ***→ thank the participant and end the interview*** |

**DOMAIN I: SATISFACTION WITH HEALTHCARE SYSTEM**

***Instructions: Now I would like to ask you some questions about healthcare system in Armenia.***

1. In general, would you say you are very satisfied, satisfied, neither satisfied nor dissatisfied, dissatisfied, or very dissatisfied with the way healthcare runs in Armenia?
2. Very satisfied
3. Satisfied
4. Neither satisfied nor dissatisfied
5. Dissatisfied
6. Very dissatisfied
7. How would you describe the situation with healthcare in Armenia? ***(Read, circle one option)***
8. It is in a state of disaster
9. It has big problems
10. It has small problems
11. It has no problems
12. It is in a perfect state

**DOMAIN II: HEALTH STATUS AND UTILIZATION OF HEALTHCARE SERVICES**

1. In general would you say your health is…
2. Excellent
3. Very good
4. Good
5. Fair
6. Poor
7. Have you used any health care services in the last 12 months?
8. Yes ***→ go to question 6***
9. No
10. What was the reason for not using health care services in the last 12 months? ***(Read, circle all that apply)***
11. There was no need ***→ go to domain III***
12. Could not afford the services ***→ go to domain III***
13. Absence of time ***→ go to domain III***
14. Do not trust doctors ***→ go to domain III***
15. Fear of the diagnosis ***→ go to domain III***
16. Other (please describe)______________________________ ***→ go to domain III***
17. Where did you receive your last healthcare services in the last 12 months?
18. Polyclinic
19. Hospital
20. At home
21. Other health care facility (please describe) ___________________
22. How did you pay for your last health care services received in the last 12 months? ***(Read, circle one option)***
23. The costs were covered by the government
24. The costs were covered out of my pocket
25. The costs were covered by private insurance
26. By the government and out of pocket
27. By private insurance and out of pocket
28. Other (please describe)________________________
29. In general, would you say you are very satisfied, satisfied, neither satisfied nor dissatisfied, dissatisfied, or very dissatisfied with the last healthcare services you received in the last 12 months?
30. Very satisfied
31. Satisfied
32. Neither satisfied nor dissatisfied
33. Dissatisfied
34. Very dissatisfied

**DOMAIN III: AWARENESS ABOUT RECENT HEALTHCARE REFORMS**

***Now I would like to ask about your opinion about the reforms in Armenian healthcare sector.***

1. Do you know that primary health care is free for all Armenian residents since 2006?
2. Yes
3. No
4. Do you know that obstetric care is free for all Armenian women since 2008? (“Obstetric Care State Certificate”)
5. Yes
6. No
7. Do you know that hospital care is free for all Armenian children under the age 7 since 2011? (“Child Health State Certificate”)
8. Yes
9. No
10. Do you know that many in-patient services are free for public employees of educational, scientific institutions of the Academy of Sciences, cultural institutions, and civil servants since 2012? (“Social package”)
11. Yes
12. No
13. Do you think that the listed reforms helped to improve access to care for Armenian citizens?
14. Yes ***→go to question 15***
15. No
16. Please explain why you think the reforms are not helpful **(*Please do not*** ***read, circle all*** ***that apply)***
17. The improvements apply to narrow population groups
18. The improvements apply to limited services
19. The improvements do not work as expected
20. Other (please describe)__________________________________________________

________________________________________________________________________________________________________________________________________________________________________________________________________________________

1. In your opinion, what (what else) should be done to improve access to health care system in Armenia?

**________________________________________________________________________________________________________________________________________________________________________________________________________________________________________________________________________________________________________________________**

**DOMAIN IV: RELATIVE IMPORTANCE OF SYSTEM CHARACTERISTICS**

**Now we would like to learn about how you value different aspects of healthcare. Please do a comparative ranking of the system characteristics according to their importance to you, by assigning each characteristic a score between 1 (the highest priority) and 7 (the lowest priority).**

1. Let’s start from identifying your first three priorities. Let me list the characteristics first (Read the characteristics with explanations). What would be your number one priority? Number 2? Number 3?

| **Characteristic** | **Rank (1-7)** |
| --- | --- |
| a. Equal access to care for all citizens |  |
| b. Modern medical equipment |  |
| c. Professional qualifications of providers |  |
| d. Respect to patients |  |
| e. Prompt attention of providers |  |
| f. Ability to choose a provider |  |
| g. Quality of basic amenities (cleanliness, ventilation, quality of food, number of patients in the room, etc. ) |  |

1. Now we would like to learn about your opinion about the above-described characteristics as applied to the current Armenian healthcare system. Using the scale of 1 - “Very bad” to 5 – “Very good”, please rate the following aspects of the healthcare system in Armenia

| **System characteristics** | **Very bad** | **Bad** | **Neither bad nor good** | **Good** | **Very good** |
| --- | --- | --- | --- | --- | --- |
| a. Equal access to care for all citizens | 􀀀1 | 􀀀2 | 􀀀3 | 􀀀4 | 􀀀5 |
| b. Modern medical equipment | 􀀀1 | 􀀀2 | 􀀀3 | 􀀀4 | 􀀀5 |
| c. Professional qualifications of providers | 􀀀1 | 􀀀2 | 􀀀3 | 􀀀4 | 􀀀5 |
| d. Respect to patients | 􀀀1 | 􀀀2 | 􀀀3 | 􀀀4 | 􀀀5 |
| e. Prompt attention of providers | 􀀀1 | 􀀀2 | 􀀀3 | 􀀀4 | 􀀀5 |
| f. Ability to choose a provider | 􀀀1 | 􀀀2 | 􀀀3 | 􀀀4 | 􀀀5 |
| g. Quality of basic amenities (cleanliness, ventilation, quality of food, number of patients in the room, etc. ) | 􀀀1 | 􀀀2 | 􀀀3 | 􀀀4 | 􀀀5 |

**DOMAIN V: GOVERNMENT’S RESPONSIBILITY FOR THE HEALTH OF INDIVIDUALS**

1. Do you think it is the individual’s responsibility to preserve his/her own health or it is the responsibility of the government? ***(Read, circle one option)***
2. Government’s responsibility
3. Individual responsibility
4. Shared, but the government’s responsibility is lager
5. Shared, but the individual’s responsibility is larger
6. Equal share of responsibility
7. Please indicate whether you agree or disagree with the following statements:

|  | ***Strongly***  ***disagree*** | ***Disagree*** | ***Neither***  ***agree nor***  ***disagree*** | ***Agree*** | ***Strongly agree*** |
| --- | --- | --- | --- | --- | --- |
| a. Government should not cover healthcare costs for those who smoke | 􀀀1 | 􀀀2 | 􀀀3 | 􀀀4 | 􀀀5 |
| b. Government should not cover healthcare costs for those who abuse alcohol | 􀀀1 | 􀀀2 | 􀀀3 | 􀀀4 | 􀀀5 |
| c. Government should not cover healthcare costs for those who abuse drugs | 􀀀1 | 􀀀2 | 􀀀3 | 􀀀4 | 􀀀5 |
| c. Government should not cover healthcare costs for those who have hereditary diseases | 􀀀1 | 􀀀2 | 􀀀3 | 􀀀4 | 􀀀5 |

1. Do you think that everyone should be required to have basic health insurance, or that it should be voluntary?
2. Health insurance should be required
3. Health insurance should be voluntary
4. In general, would you say that you tend to trust or not to trust the Armenian government?
5. Tend to trust
6. Tend not to trust
7. Don’t know/refuse to answer

**DOMAIN VI: SOVIET HEALTHCARE SYSTEM**

**Go to the first page and check the age of the respondent. Continue if the respondent is above 47 years of age. If the respondent is younger, skip to Domain VII.**

**Now I would like to ask you several questions about Soviet system of healthcare.**

1. Have you permanently resided (leaving the country only periodically and for short periods) in Soviet Armenia before 1988 (**hint for recall:** **the year of Spitak earthquake)** for at least five years?
2. Yes
3. No→ ***go to domain VII***
4. Have you used any health care services in Soviet Armenia before 1988? **(hint for recall:** **the year of Spitak earthquake)**
5. Yes
6. No ***→ go to question 25***
7. Where did you receive healthcare services in Soviet Armenia before 1988? ***(read, circle all*** ***that apply)***
8. Polyclinic
9. Hospital
10. At home
11. Other health care facility (please describe) ___________________
12. In general, would you say you were very satisfied, satisfied, neither satisfied nor dissatisfied, dissatisfied, or very dissatisfied with the way healthcare was run in Soviet Armenia?
13. Very satisfied
14. Satisfied
15. Neither satisfied nor dissatisfied
16. Dissatisfied
17. Very dissatisfied
18. How would you describe the situation with Soviet healthcare in Armenia? ***(Read, circle one option)***
19. It was in a state of disaster
20. It had big problems
21. It had small problems
22. It had no problems
23. It was in a perfect state
24. Using the scale of 1- “Very bad” to “5 – Very good”, please rate the following aspects of the Soviet healthcare system in Armenia.

| **System characteristics** | **Very bad** | **Bad** | **Neither bad nor good** | **Good** | **Very good** |
| --- | --- | --- | --- | --- | --- |
| a. Equal access to care for all citizens | 􀀀1 | 􀀀2 | 􀀀3 | 􀀀4 | 􀀀5 |
| b. Modern *(at that time)* medical equipment | 􀀀1 | 􀀀2 | 􀀀3 | 􀀀4 | 􀀀5 |
| c. Professional qualifications of providers | 􀀀1 | 􀀀2 | 􀀀3 | 􀀀4 | 􀀀5 |
| d. Respect to patients | 􀀀1 | 􀀀2 | 􀀀3 | 􀀀4 | 􀀀5 |
| e. Prompt attention of providers | 􀀀1 | 􀀀2 | 􀀀3 | 􀀀4 | 􀀀5 |
| f. Ability to choose a provider | 􀀀1 | 􀀀2 | 􀀀3 | 􀀀4 | 􀀀5 |
| g. Quality of basic amenities (cleanliness, ventilation, quality of food, number of patients in the room, etc. ) | 􀀀1 | 􀀀2 | 􀀀3 | 􀀀4 | 􀀀5 |

1. In general, how would you compare the Soviet healthcare system in Armenia with the current healthcare system in Armenia? ***(Read, circle one option)***
2. Soviet system of healthcare was better
3. Current system of healthcare is better
4. They are the same
5. In general, how would you compare the Soviet education system in Armenia with the current education system in Armenia?
6. Soviet system of education was better
7. Current system of education is better
8. They are the same
9. In general, would you say that it was easier to travel abroad in Soviet Armenia compared to the travel abroad currently?
10. Travel abroad was easier in Soviet Armenia
11. Travel abroad is easier now
12. It is the same
13. In general, how would you compare freedom of speech in Soviet Armenia with the freedom of speech in current Armenia?
14. Freedom of speech was greater in Soviet Armenia
15. Freedom of speech is greater now
16. It is the same

**DOMAIN VII: SOCIO-DEMOGRAPHIC CHARACTERISTICS**

| 1. What is your gender? | 1. Male 2. Female |
| --- | --- |
| 1. What is your completed educational level? | 1. School (8 years or less) 2. School (10 years) 3. Professional technical (10-13) 4. Institute/ University 5. Post-graduate |
| 1. Are you currently employed? | 1. Employed (including self-employed) 2. Unemployed 3. Retired 4. Student 5. Other _________________ |
| 1. What is your current marital status? | 1. Single 2. Married 3. Widowed 4. Divorced/Separated |
| 1. On average, how much does your family spend per month? | 1. Less than 50,000 drams 2. From 50,000 - 100,000 drams 3. From 100,001 - 200,000 drams 4. From 200,001 - 300,000 drams 5. Above 300,000 drams   88. Don’t know/Refuse to answer |
| 1. How many people live in your household, including you? | ___________ |
